# Removal of the Highly Toxic Anticoccidial Monensin Using Six Different Low-Cost Bio-Adsorbents

**DOI:** 10.3390/toxics12080606

**Published:** 2024-08-19

**Authors:** Samiha Hamdi, Manel Issaoui, Sonia Hammami, Ainoa Míguez-González, Raquel Cela-Dablanca, Ana Barreiro, Avelino Núñez-Delgado, Esperanza Álvarez-Rodríguez, María J. Fernández-Sanjurjo

**Affiliations:** 1 Department of Biotechnology, Faculty of Science and Technology of Sidi Bouzid, University of Kairouan, Sidi Bouzid 9100, Tunisia; samihahamdi2020@gmail.com (S.H.); manelissaoui80@gmail.com (M.I.); 2 Department of Soil Science and Agricultural Chemistry, Engineering Polytechnic School, University of Santiago de Compostela, 27002 Lugo, Spain; ainoa.miguez@rai.usc.es (A.M.-G.); raquel.dablanca@usc.es (R.C.-D.); avelino.nunez@usc.es (A.N.-D.); esperanza.alvarez@usc.es (E.Á.-R.); mf.sanjurjo@usc.es (M.J.F.-S.); 3 Laboratory of Nutrition–Functional Foods and Health (NAFS)-LR12ES05, Faculty of Medicine, University of Monastir, Avenue Avicenne, Monastir 5019, Tunisia; sonia.hammami@fmm.rnu.tn

**Keywords:** bio-adsorbents, ionophore antibiotics, monensin, natural barks, natural fibers

## Abstract

The anticoccidial monensin (MON) is a high-concern emerging pollutant. This research focused on six low-cost bio-adsorbents (alfa, cactus, and palm fibers, and acacia, eucalyptus, and zean oak barks), assessing their potential for MON removal. Batch adsorption/desorption tests were carried out, and the results were fitted to the Freundlich, Langmuir, Linear, Sips, and Temkin models. The concentrations adsorbed by the six materials were very similar when low doses of antibiotic were added, while they differed when adding MON concentrations higher than 20 µmol L^−1^ (adsorption ranging 256.98–1123.98 μmol kg^−1^). The highest adsorption corresponded to the sorbents with the most acidic pH (<5.5) and the highest organic matter and effective cation exchange capacity values (eucalyptus bark and acacia bark, reaching 92.3% and 87.8%), whereas cactus and palm fibers showed the lowest values (18.3% and 10.17%). MON desorption was below 8.5%, except for cactus and palm fibers. Temkin was the model showing the best adjustment to the experimental data, followed by the Langmuir and the Sips models. The overall results indicate that eucalyptus bark, alfa fiber, and acacia bark are efficient bio-adsorbents with potential for MON removal, retaining it when spread in environmental compartments, reducing related risks for human and environmental health.

## 1. Introduction

The global overconsumption of antibiotics has led to worldwide issues such as antibiotic resistance, underscoring the need for strict regulations and responsible medical practices [1,2,3]. Numerous classes of antibiotics used in animal husbandry are very similar or identical to those prescribed for human use [4], while others (such as the ionophore anticoccidials monensin, salinomycin, narasin, and lasalocid) are employed strictly in veterinary, as feed additives to promote growth and prevent diseases in livestock, particularly coccidiosis [5]. All these ionophores have been reported to exhibit higher toxicity than other antibiotics [6,7]. However, ionophore antibiotics have been extensively employed in farm animals, which has accounted for at least 50% of antibiotic use in the United States [8]. Consequently, several concerns related to the widespread use of these substances in cattle and poultry farming can be taken into account, raising worries due to their potential impact on human health and the environment.

After its administration, residues of the ionophore antibiotics are excreted in animal feces and urines, which are further used as fertilizer for soils [9]. In addition to animal manure/slurry, ionophores can reach soils via contaminated water and treated wastewater used for crop irrigation and through the spreading of sludge resulting from wastewater treatment that are used as fertilizers [10]. Upon reaching the soil, these emerging pollutants can impact soil bacterial communities, generating anti-microbial resistance [11].

**Figure 1:**
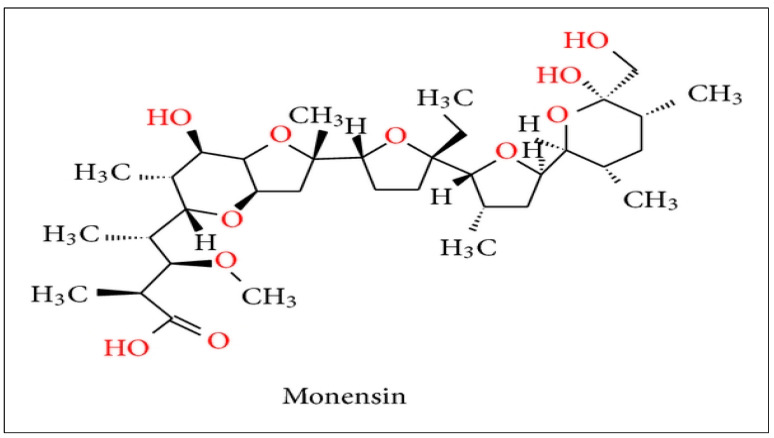
Chemical structure of monensin (MON) [13].

In the case of the ionophore antibiotic monensin (MON), due to its wide use, its potential high toxicity, and the limited knowledge with regards to its environmental repercussions, it has been classified as a high-priority environmental contaminant [12]. MON is a monocarboxylic polyether [13,14], primarily produced by the bacterium *Streptomyces cinnamonensis* [15], with its specific chemical structure shown in Figure 1.

Adsorption to soils can potentially mitigate the health risks related to the transfer of MON to crops from the soil solution [16], although limited research has been conducted in this regard. In addition, it would be needed to carry out research focused on alternatives to prevent soil and water pollution caused by this very toxic antibiotic, as well as on techniques to remediate it in already contaminated areas.

Regarding wastewater treatment, different techniques are used to remove antibiotics, like membrane separation, filtration, and advanced oxidation [17,18,19]. However, these approaches are associated with high costs and substantial waste production [20,21,22], with their application often resulting in significant energy consumption and depletion of non-renewable resources, thereby contributing to ecological impacts. Adsorption methods offer an alternative to conventional techniques for depollution, providing pollutant-removal efficiency, cost-efficiency, simplicity, and versatility [23,24], making it a promising method for pollution-remediation applications such as antibiotic remediation, especially in water. Previous studies have encouraged the use of low-cost and eco-friendly materials to adsorb antibiotics like sulfonamides present in edaphic environments [25] or tetracyclines in water [26], as they are capable of increasing the adsorption of soils with low retention capacity or to be effective in water decontamination. However, there is a lack of studies investigating the potential of eco-friendly bio-adsorbents for the removal of ionophores from water and soils. In this regard, Míguez-González et al. [27] suggested the need to perform additional research in this area, particularly focusing on advancements in the retention/removal of ionophore anticoccidials from environmental compartments, using both raw and modified bio-adsorbents as well as nanomaterials.

Natural and/or modified fiber-based materials have previously been used to remove various emerging pollutants from water systems. In this context, a study outlined by Ben Rebah and Siddeeg [28] reviewed the high efficiency of cactus fiber in removing a wide array of heavy metals, such as copper (Cu (II)) and cadmium (Cd (II)), as well as dyes like methylene blue (MB) and eriochrome black T (EBT). This aligns with data obtained in earlier research on the use of cactus fiber-based adsorbents for these purposes [29,30,31]. Prodromou and Pashalidis [32] investigated the removal of chromium (Cr (II)) using phosphorylated (with 1.5 M H_3_PO_4_) and MnO_2_-coated cactus fiber samples, comparing them to untreated cactus fiber. Additionally, recent studies have focused on using fiber materials, such as palm and alfa fibers, to remove pollutants like metals from aqueous solutions and wastewater effluents [33,34]. Other studies have explored the potential use of natural barks, such as eucalyptus and acacia barks, in water remediation. One early study evaluated a eucalyptus (*Eucalyptus camaldulensis*) bark-based composite, as new efficient adsorbent for the removal of basic blue 41 dye from aqueous solutions, showing a high level of adsorption [35]. A similar study used *Acacia raddiana* bark for the biosorption of copper cations from aqueous solutions, reporting a maximum copper biosorption capacity of 82.63 mg g^−1^ at pH 5 and a temperature of around 25–30 °C [36]. To be noted, studies on the adsorption properties of *Acacia salicina* bark are scarce, making it of interest for investigation.

With the above background, the present research was conceived as the first study simultaneously assessing alfa, cactus, and palm fibers, as well as acacia, eucalyptus, and zean oak barks, with regards to their potential for removing MON molecules from aqueous solutions. The results of this investigation could be of value in relation to controlling contamination episodes caused by this emerging pollutant, and at the same time could promote the recycling of low-cost by-products as bio-adsorbents, thus favoring sustainability, public health, and environmental protection.

## 2. Materials and Methods

### 2.1. Chemicals

MON was provided by Sigma-Aldrich (Madrid, Spain). The main physicochemical properties of this antibiotic are listed in Table S1 (Supplementary Material). Acetonitrile (purity ≥ 99.9%), and phosphoric acid (85% extra pure) were from Fisher Scientific (Madrid, Spain), while 95% pure CaCl_2_ was from Panreac (Barcelona, Spain). In addition, optima-grade reagents methanol, CaCl_2_, acetic acid, Trichloroacetic (TCA), and 2,4-Dinitrophenol (DNP) acids were purchased from Sigma-Aldrich (Madrid, Spain). For HPLC analyses, all necessary solutions were prepared with milliQ water obtained from Millipore (Madrid, Spain).

### 2.2. Bio-Adsorbent Materials

Six bio-adsorbents were used: (i) three natural fibers: alfa fiber (derived from *Stipa tenacissima*, a plant frequently distributed in central and southern Tunisia), which was sampled from the Hadej region (Menzel Bouzaiane, Sidi Bouzid, Central Tunisia); palm fiber (*Phœnix dactylifera* L.), which was sampled from the Midass region (Tozeur, southern Tunisia); and cactus fiber (*Opuntia ficus-indica*), from the Tala region (Kasserine, North-central Tunisia); (ii) three natural barks: two of them were acacia and eucalyptus barks, which were from the tree species *Acacia salicina* and *Eucalyptus camaldulensis*, respectively, both commonly found in arid and semi-arid regions of Tunisia, and that for this study were obtained from the Maknessy region in Sidi Bouzid, central Tunisia; and the third bark sample was derived from the zean oak tree (*Quercus canariensis* Willd), collected from the Tabarka region in northwestern Tunisia, where it is commonly distributed.

The sampling of the bio-adsorbent materials was done in March 2023 from different Tunisian locations, and they were subsequently transferred to the laboratory for preparation and physicochemical analyses. Before further processing, the bio-adsorbent samples were washed and dried (in an oven at 60 °C for 24 h), then crushed using an automatic grinder (SCP SCIENCE SP-2000 Swing Mill Grinder). After crushing, the bio-adsorbents used in the experiment were sieved through a 100-µm mesh.

### 2.3. Characterization of the Bio-Adsorbents

The six bio-adsorbents were characterized before performing adsorption-desorption tests. The physicochemical parameters that were assessed were pH and electrical conductivity (pH_w_ and EC, respectively) measured in water, pH in 0.1 M KCl solution (pH_KCl_), pH of the point of zero charge (pH_PZC)_, humidity (H%), bulk density (D, expressed in g cm^−3^), swelling index (SI%), porosity (P%), ash (As) content, organic matter content, exchangeable cations (Ca_e_, Mg_e_, Na_e_, K_e_, and Al_e_, expressed in cmol_c_ kg^−1^), and effective cation exchange capacity (eCEC, also expressed in cmol_c_ kg^−1^). The methods employed for the characterization of the bio-adsorbents are detailed in the Supplementary Material, where references to the methods presented in Fox and Kamprath [37], Lopes et al. [38], Nebot et al. [39], Peech [40], and Rodríguez-López et al. [41] are included.

### 2.4. Experimental Design

#### 2.4.1. Influence of Environmental Factors

The main factors considered were adsorbent weight, contact time, and MON concentration. These factors were selected taking into consideration previous kinetic studies on ionophore antibiotics such as monensin and lasalocid [6], as well as non-ionophore antibiotics such as amoxicillin [42], when adsorbed onto soils and different bio-adsorbents, which had indicated that 48 h were sufficient to achieve equilibrium in the adsorption process. Additionally, the bio-adsorbent mass was fixed as 0.5 g, which were added to 10 mL of the MON solutions, with the samples being shaken under dark conditions to prevent photodegradation, particularly under ultraviolet (UV) light, which can impact the stability of MON molecules.

All the experiments were conducted at room temperature (25 ± 2 °C) without adjusting the pH, which is relevant to many real-world sorption applications, especially in environmental remediation. In addition, standard calibration procedures were performed before measuring with the pH-meter and atomic absorption spectrophotometer.

#### 2.4.2. Experiments on Adsorption and Desorption (Batch Tests)

Batch experiments were employed to conduct adsorption and desorption investigations across the entire array of bio-adsorbents, following the procedure detailed in the Supplementary Material. Moreover, details about the experimental conditions for the adsorption-desorption tests were briefly mentioned in the Supplementary Material. Adsorption and desorption studies were executed through batch experiments, wherein 0.5 g of adsorbent were immersed in 10 mL of MON solutions using six concentrations, ranging from 5 to 100 μmol L^−1^ in 0.005 M CaCl_2_ solutions, as done previously in studies for tetracycline and sulfadiazine antibiotics in natural and modified clays [43,44] and onto forest bio-adsorbents like pine bark and oak ash [45]. CaCl_2_ was used as a background electrolyte to maintain constant ionic strength. The shaking time was 48 h, which was found to be a sufficient duration to achieve equilibrium, as determined in previous unpublished kinetic studies. The desorption tests involved the addition of 10 mL of 0.005 M CaCl_2_ solutions, followed by the application of the same procedure as employed in the adsorption tests. All these experiments were conducted in triplicate.

#### 2.4.3. Quantification of MON

Prior to the quantification analysis, certain procedural steps were considered necessary to enhance the detectability of the MON antibiotic (details are provided in the Supplementary Material). MON quantification for adsorption and desorption phases was performed in triplicate, at room temperature (25 ± 2 °C), and with unmodified pH, using an UltiMate 3000 HPLC liquid chromatograph (Thermo Fisher Scientific, Madrid, Spain). During the quantification process, all HPLC samples from the adsorption-desorption steps were run with an isocratic method, with a single phase composed of methanol (88.5%), water (10%), and acetic acid (1.5%), with a flow rate set at 1 mL min^−1^. Subsequently, the obtained data were analyzed using Chromeleon software version 7 (Thermo Fisher Scientific, Madrid, Spain). Further details concerning the HPLC equipment are outlined in the Supplementary Material. For the separation of MON, the following conditions were used: the injection volume for analysis was 200 µL, the total analysis time was 35 min, with a wavelength of 392 nm. The MON peak appeared divided into three peaks at times: 6.9 min, 7.2 min, and 8.4 min. Then, the areas of these three peaks were summed up. It is stressed to note that between each measurement, the syringe was rinsed with the running solution. Figure S1 (Supplementary Material) presents some example chromatograms. Finally, taking into account the MON concentrations added, minus the equilibrium concentrations (C_eq_; µmol L^−1^), allows the calculation of the amounts of MON adsorbed.

### 2.5. Calculation and Statistical Treatment

The experimental data obtained in the batch adsorption tests were adjusted to the Freundlich (Equation (1)), Langmuir (Equation (2)), Linear (Equation (3)), Sips (Equation (4)), and Temkin (Equation (5)) models:

q_a_ = K_F_ ∗ C_eq_^n^, (1)

q_a_ = (q_m_ K_L_ ∗ C_eq_)/(1 + K_L_ ∗ C_eq_), (2)

q_a_ = K_d_ ∗ C_eq_, (3)

q_a_ = q_m_ ∗ ((K_S_ ∗ C_eq_)^n^/(1 + (K_S_ ∗ C_eq_)^n^), (4)

q_a_ = β lnK_T_ + β lnC_eq_, (5)

where q_a_ (µmol kg^−1^) is the quantity of antibiotic retained by the different bio-adsorbents at equilibrium, the concentration of antibiotic present in the solution at equilibrium is denoted as C_eq_ (µmol L^−1^); K_F_ is the Freundlich parameter associated with adsorption capacity (L^n^ μmol^1−n^ kg^−1^); n (dimensionless) is the Freundlich linearity index, K_L_ is the Langmuir adsorption constant (L μmol^−1^), while qm is the maximum adsorption capacity according to the Langmuir model (μmol kg^−1^). K_d_ (L kg^−1^) is the distribution coefficient in the linear model; K_S_ represents the Sips adsorption constant, indicating the affinity of the adsorbate for the surface (L µmol^−1^), while n (dimensionless) reflects the heterogeneity of the equilibrium system. In the Temkin model, β = RT/bt, bt is the Temkin constant associated with sorption (J/mol), R is the universal gas constant [46,47], and T denotes the temperature at 25 °C (K = 298 °C). Additionally, K_T_ represents the Temkin isotherm equilibrium binding constant (L g^−1^).

In the current work, the hysteresis index (HI) (Equation (6)) was calculated using the formula established in prior literature [41]:

HI = (q_a_^D^ − qa^S^)/q_a_^S^, (6)

where q_a_^S^ represents the adsorption concentrations of MON in the studied bio-adsorbents and q_a_^D^ denotes the final concentration after the desorption experiments.

The adjustment of adsorption experiments to the different statistical models, along with one way-ANOVA analysis, was conducted using IBM SPSS Statistics version 21 software (New York, NY, USA).

In order to achieve more comprehensive information about the affinity of binding sites and to analyze the results of adsorption modeling, the Scatchard plot analysis [48], a widely used technique, also known as the independent-site oriented model, was applied to the experimental data. Compared to other mathematical transformations of the classical Langmuir equation, awareness about the equilibrium concentration ranges where the Langmuir model shows good fit to the experimental data can be acquired more easily through the Scatchard equation, which is represented as follows:

q_a_/C_eq_ = Q_m_^S^ K_b_ − q_a_ K_b_, (7)

where q_a_ and C_eq_ have the same meaning as mentioned above, and Q_m_^S^ and K_b_ are the Scatchard parameters, with Q_m_^S^ (expressed in µmol kg^−1^) being the theoretical saturation capacity (also known as a parameter related to the number of binding sites involved in a particular sorption process), whereas K_b_ is considered as a constant related to the affinity between sorbent and sorbate (also known as binding constant). Additionally, the Scatchard model was used to analyze adsorption data by plotting q_a_/C_eq_ against q_a_, creating a Scatchard plot (Figure S2, Supplementary Material). The shape of the plots obtained indicates: (i) a straight line reveals uniform adsorption sites; (ii) a nonlinear curve suggests nonspecific or multiple interactions; and (iii) concave curves denote negative cooperative effects or heterogeneous sites, while convex curves imply positive cooperative effects [44,49]. Deviations from linearity (as determined by R^2^ values) can signal non-specific or multi-type interactions between adsorbents and adsorbates [50].

All the above indicated methods suppose the first steps included in a wide research program, with a series of subsequent phases to be accomplished with regards to empirical and computational tasks, according to previously defined protocols [51,52].

## 3. Results

### 3.1. Bio-Adsorbents Characteristics

Table 1 shows the values corresponding to the physicochemical parameters determined for the six bio-adsorbents studied.

As shown in Table 1, the pH values (in water) ranged between 5.1 and 7.4 for the fibers, while the range was 4.9–5.7 for the bark samples. These values were higher than those of pH_KCl_ for alfa fiber, as well as for acacia and eucalyptus barks, which ranged from 4.2 to 5.1. Conversely, the pH_w_ values were lower than the pH_KCl_ values for cactus and palm fibers, as well as for zean oak bark, which ranged from 4.8 to 7.6. The pH_PZC_ values shown in Table 1 were estimated from the intersection between the bisector line and the graphical representation of pH_final_ versus pH_initial_ (see details in the Supplementary Material) as shown in Figure S1 (Supplementary Material). The pH_PZC_ values of the different bio-adsorbents here studied were in the range of 4.3–7.4, as indicated in Table 1 and Figure S3 (Supplementary Material). It is crucial to bear in mind that at pH < pH_PZC_, the adsorbent surface is positively charged, and the adsorption of anions is consequently favored (as observed for acacia and eucalyptus barks, and also for alfa fiber), whereas, at pH > pH_PZC_, the biosorbent surface is negatively charged, and biosorption of cations is favored (as noted for cactus and palm fibers along with zean oak bark) [53,54,55]. The fibers had the highest EC levels (ranging 21–818 dS m^−1^), being lower for the barks (4.2–19.7 dS m^−1^). The moisture content (H%) values were typically higher (ranging 9.3–11.5%) for alfa fiber and eucalyptus and acacia barks, compared to those observed for cactus and palm fibers, as well as for zean oak bark (ranging 5.4–7.0%) (Table 1). Thus, the highest values for dry matter (DM) content were observed for samples with lower H% scores, specifically cactus (93.8%) and palm (94.6%) fibers, as well as oak bark (93.0%) (Table 1).

Concerning the bio-adsorbent’s porosity, their values oscillated between 41.6% and 86.6% for alfa fiber and palm fiber, respectively. The bark samples presented low levels of ash (As) content (1.22, 1.42, and 1.91% for acacia, eucalyptus, and zean oak bark, respectively) and similar volatile matter (VM%) content (Table 1).

The bulk density (BD) values of the studied bio-adsorbents varied between 0.81 g cm^−3^ of cactus fiber and 1.53 g cm^−3^ of acacia bark, which were lower than the real density (RD) scores (ranging 1.14–1.97 g cm^−3^) (Table 1).

Regarding the swelling indices (SI), acacia and eucalyptus barks and alfa fiber had the highest swelling power, compared to the other adsorbent materials, and are also the ones with the highest density (Table 1).

The organic matter content (OM) also shows a marked variability, oscillating between 18.84% (palm fiber) and 50.25% (eucalyptus bark). In the current study, eucalyptus and acacia barks, together with alfa fiber, present the highest OM values (>40%), whereas it is below 30% for the other bio-adsorbents. Similarly, eucalyptus and acacia barks, along with alfa fiber, exhibited the highest organic carbon (OC%) values, oscillating between 24.12% (acacia bark) and 19.54% (alfa fiber), while the OC% values for the other adsorbent materials did not exceed 14.32%. Among the exchangeable cations, Ca_e_ was predominant in alfa fiber and in the three barks, while Mg_e_ predominated in cactus and palm fibers. The highest Na_e_ values were observed in eucalyptus and acacia barks, together with alfa fiber. Conversely, Al_e_ showed low levels for all the bio-adsorbents (ranging between 0.05 and 0.18 cmol_c_ kg^−1^), with its highest values associated with acacia and eucalyptus barks, and with alfa fiber, coinciding with its lower pH. Furthermore, both eucalyptus and acacia barks, as well as alfa fiber, showed the highest eCEC scores. Note that the three bio-adsorbents with the lowest OM contents (palm fiber, cactus fiber, and zean oak bark) are those with the lowest eECE values, which is indicative of the importance of OM in the generation of electrical charges.

The wet sieving analysis indicated that the studied bio-adsorbents had particle sizes mostly ranging from 75 to 100 µm (0.075 to 0.1 mm) for fiber samples and from 50 to 75 µm (0.05 to 0.075 mm) for bark samples. All samples were well-homogenized before being used in this investigation.

### 3.2. MON Adsorption

**Figure 2:**
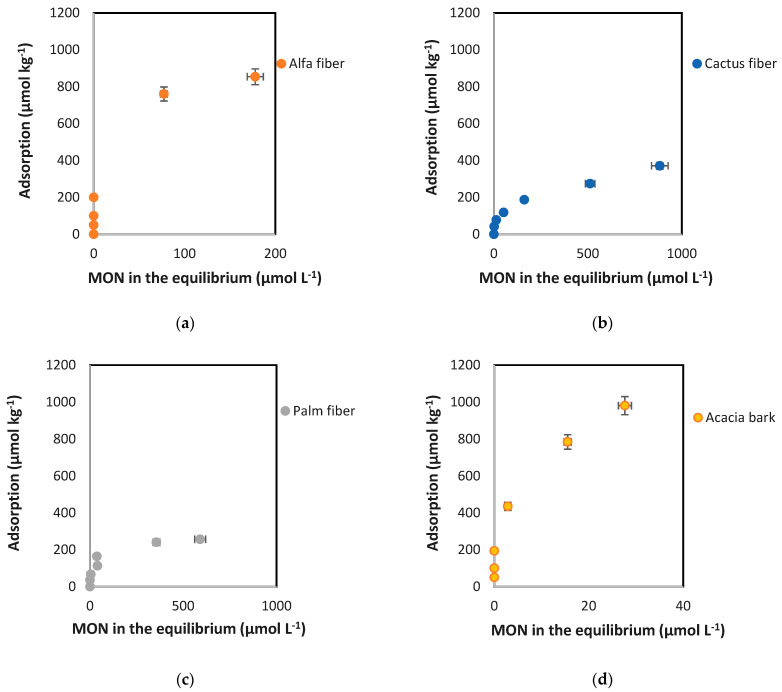

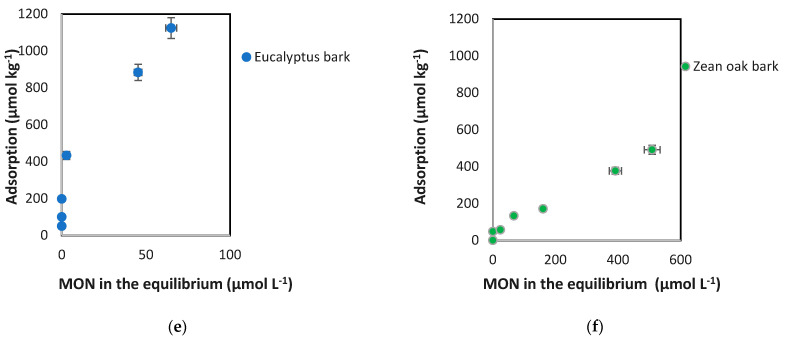
Adsorption curves for MON corresponding to the different bio-adsorbents used: natural fibers (**a**–**c**) and barks (**d**–**f**). Average values (n = 3), with coefficients of variation always <5%. When the error bars are not visible, it means that they are smaller than the symbols. Adsorption tests conditions: 0.5 g of adsorbent with 10 mL of 0.005 M CaCl_2_ solutions containing from 5 to 100 µmol L^−1^ of MON, shaking for 48 h at 50 rpm in the dark and at 25 ± 2 °C, then centrifuging (4000*× g*) and filtering by 0.45 µm before HPLC quantification.

Figure 2 shows adsorption curves, plotting the amount of antibiotic adsorbed (q_a_, in µmol kg^−1^) versus its concentration in the equilibrium solution (C_eq_, in µmol L^−1^). As depicted in Figure 2, the adsorbed amounts increase with the rise in equilibrium concentration (C_eq_), while the slopes gradually decrease, with the most pronounced decrease being for cactus and palm fibers.

**Figure 3:**
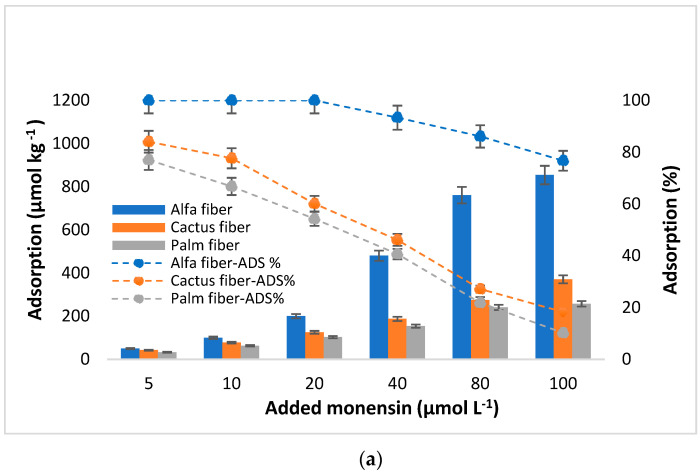

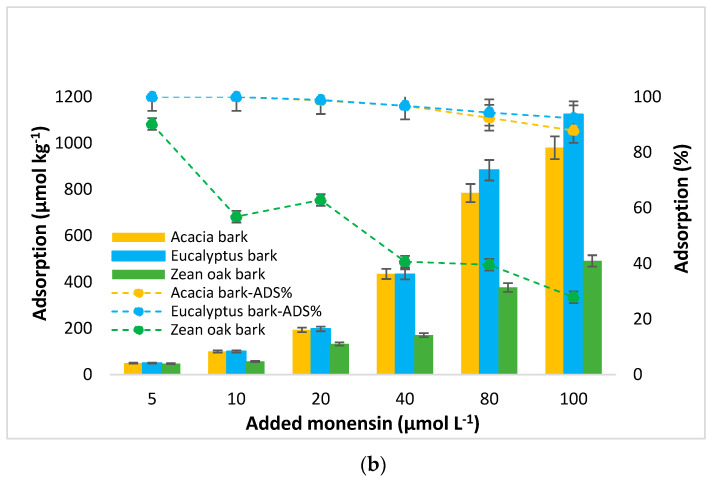
Monensin (MON) adsorption of (in µmol kg^−1^ and %) onto natural fibers (**a**) and barks (**b**), as a function of the concentration of the antibiotic added (µmol L^−1^). Average values (n = 3), with coefficients of variation always <5%. When the error bars are not visible, it means that they are smaller than the symbols.

Additionally, the adsorption capacities of the studied bio-adsorbents, expressed in µmol kg^−1^ and as a percentage, are shown in Figure 3. According to these data, the maximum adsorption corresponded to eucalyptus bark, followed by acacia bark and alfa fiber. Specifically, for the highest concentration of MON added (100 µmol L^−1^), the adsorbent amounts were 1123.98, 930.34, and 853.98 µmol kg^−1^, for eucalyptus and acacia barks, and for alfa fiber, respectively. Contrary, for the same added concentration the minimum adsorption amounts were observed for palm fiber (256.98 µmol kg^−1^), followed by cactus fiber (370.98 µmol kg^−1^), and then zean oak bark (491.18 µmol kg^−1^).

Considering the adsorption data presented in Figure 3, it is crucial to note that the amounts of MON adsorbed increase as a function of the concentration of antibiotic added, contrary to the adsorption percentages, which decrease with the rise of the MON concentration added, especially in case of palm and cactus fiber, and of zean oak bark. Adsorption percentages are close or equal to 100% for eucalyptus and acacia barks, as well as for alfa fiber, when the MON concentrations added ranged between 5 and 20 µmol L^−1^, while the scores decreased for cactus and palm fibers and for zean oak bark, going from 84.0 to 47.8% (Figure 3).

### 3.3. Fitting of Experimental Data to Adsorption Models

The details corresponding to the fitting of MON adsorption experimental data to the Freundlich, Langmuir, Linear, Sips and Temkin models are presented in Table 2.

### 3.4. MON Desorption

**Figure 4:**
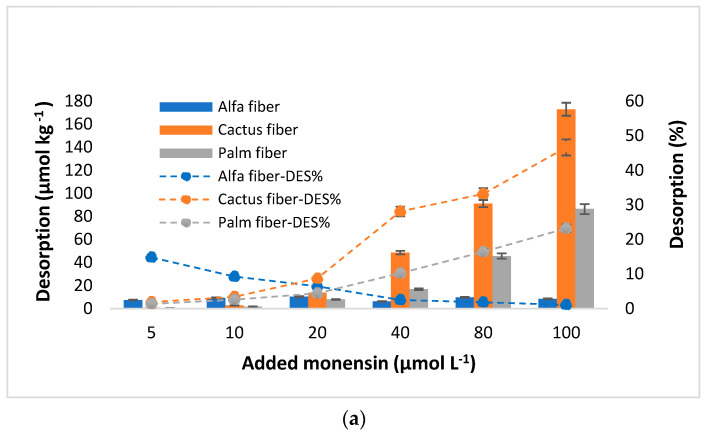

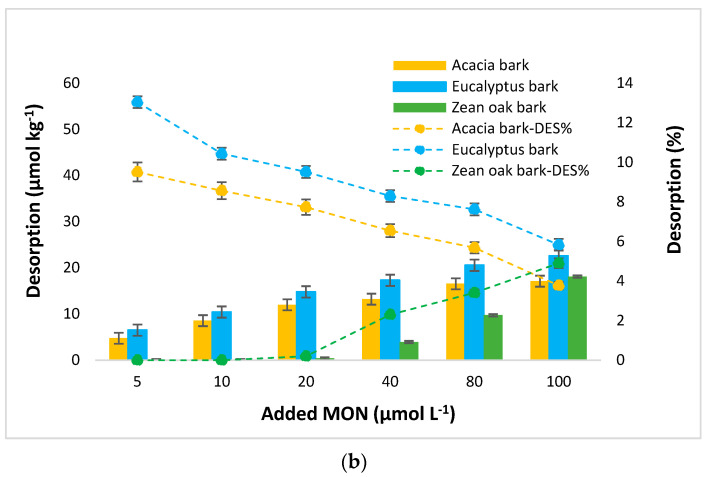
Desorption of monensin (MON) (in µmol kg^−1^ and %) from natural fibers (**a**) and barks (**b**), as a function of the concentration of the antibiotic added (µmol L^−1^). Average values (n = 3), with coefficients of variation always <5%. When the error bars are not visible, it means that they are smaller than the symbols.

Figure 4 presents the amounts of MON desorbed from the different bio-adsorbents, as well as the desorption percentages, versus the initial MON concentrations added (µmol L^−1^). When the MON concentrations added are lower than 20 µmol L^−1^, the amounts desorbed are generally low (<10%) and similar for all the bio-adsorbents. At higher concentrations added, clearly higher desorption scores are observed for cactus fiber and palm fiber (Figure 4).

For cactus fiber the desorbed quantities reach 173 µmol kg^−1^ when the added concentration is 100 µmol L^−1^, which corresponds to almost 47% of the added antibiotic. As for palm fiber, the maximum desorption value was 84.4 µmol kg^−1^ (23.2%), also associated with the highest dose added. It is important to note that, at the three lowest MON concentrations added (5, 10, and 20 µmol L^−1^), the desorption percentages observed for both cactus and palm fibers did not exceed 9% and 5%, respectively. Alfa fiber exhibited the lowest desorption of MON (8.45 µmol kg^−1^), representing 1.1% of the added antibiotic, at a MON concentration added of 100 µmol L^−1^ (Figure 4). The desorbed amounts never exceed 23 µmol kg^−1^ for eucalyptus and acacia barks, remaining below 10% across the four highest concentrations of antibiotic added (from 20 to 100 µmol L^−1^). Regarding zean oak bark, it did not desorb MON at the three lowest concentrations added, while it began to desorb when the initial concentration reached 40 µmol L^−1^, although it did not exceed 5% in any case (Figure 4). Thus, the desorption sequence for the three highest concentrations of antibiotic added was: alfa fiber < zean oak bark < acacia bark < eucalyptus bark < palm fiber < cactus fiber.

The MON desorption percentages obtained for most of the here-studied sorbent materials demonstrate the low reversibility of the adsorption process. The calculation of the hysteresis index (HI) supports this idea, obtaining values greater than 0.906 in most samples, except in cactus and palm fibers, with average values around 0.235 and 0.429, respectively (Table 3).

Table 4 shows that, as happened regarding the fitting of the adsorption data, the desorption experimental results were well-described by both the Temkin model (with R^2^ values ranging from 0.984 to 1.00) and the Sips model (R^2^ ranging from 0.918 to 0.995).

## 4. Discussion

### 4.1. MON Adsorption

Most of the adsorption curves included in Figure 2 are L-type, according to Giles et al. [56], while those obtained for alfa fiber and, especially, for eucalyptus bark and acacia bark, can be considered type H, which are a special case of L-type curves, indicating that the adsorbent surface has a high affinity for the solute [57]. A decreasing slope with increasing concentration is indicative of this type of curve and is explained by the decrease in adsorption sites available on the adsorbent [58]. Generally, these curves exhibit non-linearity and concavity, suggesting that at low C_eq_ values there is a strong affinity for the bio-adsorbents, resulting in most of the pollutant being adsorbed in almost all the samples. It is important to note that in the case of zean oak bark adsorption curves have a higher tendency to linearity, although they can also be considered type L, but with a much lower slope compared to those of alfa fiber and both acacia and eucalyptus barks.

As shown in Figure 3, for added concentrations ranging from 40 to 100 µmol L⁻^1^, a decrease in the adsorption percentages is evidenced, which would be due to the adsorption sites in the bio-adsorbents gradually becoming saturated as higher concentrations of antibiotic are added [59]. Note that the percentages remain high (>90%) for acacia and eucalyptus barks, and for alfa fiber (>76.6%). These adsorption percentages indicate the strong affinity of the antibiotic for acacia and eucalyptus barks, and for alfa fiber, at all the concentrations added (with mean values of 92.6%, 95.9%, and 97.0%, respectively), while the other bio-adsorbents show percentages lower than 40% from 40 µmol L^−1^ of antibiotic added. In relation to previous studies dealing with MON adsorption, Sassman and Lee [6] indicated that MON has the potential to be adsorbed on soils of varying physicochemical composition, with and without manure amendment, and the analysis of drainage water indicated that soil attenuation post-land application would significantly decrease the amount of MON entering the surface water.

From a structural perspective, carboxylic ionophores such as MON are aliphatic chains that bear five cyclic ether rings, with a carboxylic group on one end and with one or more hydroxyl groups on the other end [60] (Figure 1). Overall, the specific adsorption behavior of this ionophore antibiotic is influenced by the type and arrangement of these functional groups within its chemical structure, as well as by the properties of the adsorbent surface. According to the literature, it is assumed that ionophores are generally found in different environmental compartments (soil, water, and sediment), at a wide range of concentrations [16,61,62]. Several authors have found MON in surface waters, such as Bak and Björklund [16], who reported mean concentrations around 20 ng L^−1^, or in streams of the southern Pampas, Argentina [62]. Hussain et al. [15] indicate that the persistence of MON in surface water was primarily dependent on the pH values in the affected environment and on its acidic pK_a_ values. Hafner et al. [11] reported the transport of MON to shallow groundwater after irrigation with dairy lagoon water. Bak and Björklund [16] detected the presence of MON molecules in soils at a concentration of 8 µg kg^−1^. Although there are few studies on the uptake of this antibiotic by crops, Hilaire et al. [63] reported it for grassland species. This contaminant can further pass through the food chain to animals and humans.

Soil parameters such as pH, organic matter, or eCEC have been indicated to be of fundamental relevance in the behavior and fate of antibiotics once they are released into the environment [64,65]. Furthermore, the high values of porosity, moisture content, and swelling indices can enhance the adsorption capacities of materials used as antibiotic adsorbents [43,66]. In the current work, the highest adsorption efficiency corresponded to the sorbents that had a lower pH (Figure S4, Supplementary Material) and ash content (Table 1), higher OM, porosity, SI, H%, and eCEC levels, and more exchangeable Ca and Na (alfa fiber, acacia bark, and eucalyptus bark) (Table 1). The OM present at high percentages in all the studied bio-adsorbents, at the pH values of these materials (between 4.9 and 7.4), will mainly present a negative charge, mostly in their carboxylic groups, that have an acidic pK_a_, which can ionize, forming carboxylate ions (RCOO⁻) in aqueous solutions. In relation to the electrical charge of MON, most of the experiments that have been carried out dealing with pK_a_ calculation have been performed in organic solvents or in solvent/water mixtures, giving a pK_a_ = 6.4–6.7, but these results would be difficult to apply to aqueous media [60]; in this sense, the authors of the latter research obtained a pK_a_ value = 4.5 in water, and, considering this pH and those of the bio-adsorbents (all above 4.5), MON would tend to become negatively charged, with which it could join the organic radicals of the positively charged bio-adsorbents; however, the binding to carboxylic groups would be carried out through a cationic bridge, which could be a frequent mode of interaction between MON and organic groups, as noted by Hansima et al. [67]. In relation to this, Ca^2+^ is known for its implication in the adsorption process where it can act as a bridge between the adsorbent surface and absorbates such as antibiotics [68]. In addition to that commented for Ca^2+^, several authors have indicated that MON has a high affinity for Na^+^ [6,69]. Sun et al. [60] confirmed that the complexation of MON with Na^+^ (Figure S5, Supplementary Material) is approximately one order of magnitude more favorable than with potassium ions, both in water and in methanol. The higher exchangeable Na^+^ (and Ca^2+^) contents of some of the sorbents used in the current research (alfa fiber, acacia bark, and eucalyptus bark) would contribute to justify their sorption capacity.

In view of the above, MON adsorption onto the studied bio-adsorbents could take place through different mechanisms, which could act simultaneously. One of the mechanisms is electrostatic attraction between the negative charges generated on the surface of the antibiotic at pH > pK_a_ and the positive ones that appear in certain protonated amine groups (-NH_3_^+^) of the abundant organic matter present in all the bio-adsorbents under study. In fact, positive charges would be more relevant in those bio-adsorbents having higher organic matter contents and lower pH values, such as acacia and eucalyptus bark, and alfa fibers (Table 1). These three bio-adsorbents also show the highest acidity considering the pH in the equilibrium solution of the adsorption process (Table S2, Supplementary Material).

Another adsorption mechanism would make use of a cationic bridge (especially using Na^+^ and Ca^2+^) between the negative charges of the antibiotic and the negative charges that appear at pH values above 5 in certain organic functional groups such as carboxylic acids (-COO^−^). All this justifies that the three bio-adsorbents with more acidic pH, more organic matter and eCEC levels, and more exchangeable Na^+^ and Ca^2+^ (and less K^+^ and Mg^2+^) are the most effective at retaining MON, specifically adsorbing more than 76.5% of the amount added, even when using the highest antibiotic concentrations. In addition, Hansima et al. [67] indicate that MON has a hydrophobic nature and a great tendency to form colloidal bonds (considering soil environments), the main adsorption mechanisms being cation bridging, metal complexation, and hydrophobic interactions with OM.

Regarding the current research, other types of interactions that are possible involve hydrogen bonds between different oxygen-bearing functional groups, such as the ether groups (-O-) of MON and phenolic or carboxylic functional groups of bio-adsorbents.

The scarcity of previous research on MON adsorption onto biomaterials like forest bio-adsorbents complicates comparisons with the current study. Alternatively, and dealing with edaphic environments, Hussain and Prasher [70] assessed MON adsorption on sandy clay loam, and sandy soils, under varying pH conditions and organic matter contents, also finding greater MON affinity for soils with lower pH and higher organic matter content. Furthermore, several studies that used bio-adsorbents and different pollutants, such as eucalyptus bark powder for dyes [71] or palm fiber for cephalexin [72], mentioned the role of aromatic compounds on their adsorption capacities forward contaminants. Additionally, tannins present in bio-adsorbents derived from trees and plants can be important for the adsorption process, helping in establishing bindings between pollutants and adsorbent surfaces [73].

### 4.2. Fitting to Adsorption Models

In the current research, for all the tested bio-adsorbents, the Temkin and Langmuir models fit well the experimental adsorption data (with R^2^ ≥ 0.968 and ≥0.934, respectively), while the Sips model shows a somehow poorer fit (R^2^ ≥ 0.869). In the case of the Freundlich model the value was R^2^ ≥ 0.723 is obtained, whereas the worst corresponded to the Linear model (0.578 ≤ R^2^ ≤ 0.634) (Table 2).

The fact that the Temkin model gives the best fitting for all the bio-adsorbents here studied would suggest that adsorption is taking place mainly by means of electrostatic attractions between charges of different signs of the antibiotic and the bio-adsorbents [2], which underscores the significance of chemisorption processes [74]. Moreover, Table 2 shows that the highest Kt values (oscillating between 0.223 and 4.219 L g^−1^) and bt values (ranging between 0.231 and 6.765 J/mol), corresponded to eucalyptus and acacia barks, followed by alfa fiber, which imply a more efficient adsorption process and a stronger affinity between these bio-adsorbents and the pollutant (adsorption energetically favorable). However, the lower Kt and bt values observed for both cactus and palm fibers, which have the lowest adsorption, compared to the other bio-adsorbents, suggest lower interaction between the adsorbate molecules and these adsorbents. Generally, the fitting of adsorption data to the Temkin model shows a linear decrease in adsorption energy with surface occupation, which is related to adsorbent-adsorbate interactions [75].

Regarding the Langmuir model, the maximum adsorption capacity (q_m_) was 1046.1 µmol kg^−1^ (for eucalyptus bark) (Table 2), which was in agreement with the measured data (1123.9 µmol kg^−1^ for eucalyptus bark) (Figure 2 and Figure 3). Acacia bark and alfa fiber also show high q_m_ scores (>950 µmol kg^−1^), with the lowest value obtained for zean oak bark (527.9 µmol kg^−1^), in agreement with the amounts adsorbed in the experiment. With regards to K_L_ (the constant related to the affinity of the binding sites and energy of adsorption [76]), its highest values were associated to eucalyptus and acacia barks, as well as to alfa fiber (0.319, 0.276, and 0.149 L kg^−1^, respectively) (Table 2), suggesting that there is a high affinity between these bio-adsorbents and MON. In this regard, the K_L_ values for these materials were higher than those reported in a previous investigation conducted by Mirizadeh et al. [77], who studied the adsorption of other antibiotics like tetracycline and ciprofloxacin using raw palm waste as adsorbent. In the current research, both cactus and palm fibers, along with zean oak bark, had lower K_L_ values, around 0.069, 0.043, and 0.082 L kg^−1^, which are consistent with those obtained for oak ash and pine bark referred to other antibiotics like ciprofloxacin (K_L_ = 0.05 L kg^−1^) and trimethoprim (K_L_ = 0.03 L kg^−1^) [78]. Existing a good fit of experimental data to the Langmuir model, in such cases the adsorption process appears to be dominated by chemical and monolayer adsorption on a surface, featuring a finite number of identical and energetically equal sites, which would explain the decrease in the adsorption percentage as the added concentration increases [79,80], being a chemical adsorption mechanism primarily influenced by strong π–π interactions through electrostatic attraction and physical retention [81] and leading to more effective MON adsorption onto the bio-adsorbents.

Concerning the Sips model, the values of the Sip adsorption constant (K_S_), which is related to the affinity of the adsorbate towards the adsorbent surface [49], ranged from 0.187 to 6.201 L kg^−1^, with the highest scores found for eucalyptus bark, acacia bark, and alfa fiber (Table 2). The n parameter of the Sips model typically indicates the degree of heterogeneity in the adsorption system. When n is equal to 1, the Sips isotherm returns to the Langmuir isotherm, predicting homogeneous adsorption. On the other hand, the deviation of the n value from 1 approximates the fit to a Freundlich isotherm, indicating interactions with heterogeneous surfaces [49,82]. In the current research, n ranged between 0.173 and 2.754, with the highest values (greater than 1) corresponding, again, to eucalyptus bark, acacia bark, and alfa fiber. Values of n greater than 1 would indicate that the adsorbed molecules have a strong affinity towards adsorbent sites [83], and this would coincide with the greater adsorption capacity of these three bio-adsorbents.

In the Freundlich model, the linearity index (n) can be seen as indicative of the reactivity of the active sites in the adsorbent [84]. Values of n greater than 1 would correspond to sites of high adsorption energy, with high accessibility of the antibiotic to the surface of the adsorbent [84,85]. It is shown that n > 1 for eucalyptus bark, acacia bark, and alfa fiber (2.68, 2.48, and 1.41, respectively). However, for the rest of the materials (cactus fiber, palm fiber, and zean oak bark), the values of n are clearly lower than 1 (0.21 to 0.45), which would indicate that there is a limitation in the specific adsorption sites available on the surface of the sorbents. This would be related to a non-linear and concave adsorption curve (Figure 2), evidencing the greater difficulty in adsorption as the antibiotic concentration increases, because the high-energy sites are those that are occupied first [86,87]. On the other hand, the Freundlich constant, K_F_, related to the degree of interaction between the antibiotic and the adsorbents (the higher this value, the higher the adsorption intensity) [88], presents the following sequence: eucalyptus bark > acacia bark > alfa fiber > zean oak bark > cactus fiber > palm fiber. This sequence agrees with the adsorption results obtained for the different bio-adsorbents (Figure 3).

### 4.3. Scatchard Plots Analysis

Considering the sorption of antibiotic molecules onto various adsorbents, it is well-known that bio-adsorbent materials can interact with antibiotic molecules through multiple mechanisms, such as ion exchange, hydrogen bonding, and complex formation. Table S3 (Supplementary Material) shows the results of Scatchard parameters and plots. Also, the Scatchard plots obtained for the six bio-adsorbents under investigation are shown in Figure S2 (Supplementary Material). These kinds of plots are typically used to assess receptor affinity for ligands, identify the number of binding sites, and calculate binding constants (K_b_) [50]. R^2^ values across the data range may indicate nonspecific or multi-type interactions between adsorbate molecules and surface sites. The overall R^2^ values were used to discuss the results, with R^2^ (L) and R^2^ (H) values noted on the Scatchard plots (Figure S2, Supplementary Material). In the current work, the R^2^ values calculated were always higher than 0.805 (except for the cactus and palm fibers, with R^2^ equal to 0.7 and 0.61, respectively), which indicates that the presence of nonspecific interactions is higher for most of the sorbent materials than for the cactus and palm fibers, being the highest the ones obtained for eucalyptus and acacia barks (0.968 and 0.960, respectively) and for alfa fiber (0.952). In addition, the Scatchard plots obtained for cactus and palm fibers, as well as for zean oak bark, can be considered as concave curves that are associated with a negative cooperative adsorption phenomenon, as well as to surface heterogeneity [89,90]. In contrast, for acacia and eucalyptus barks, and alfa fiber, the showed curves were considered as convex, indicating positive cooperative phenomena, meaning that initial adsorption occurs with low affinity, but the adsorbate becomes a likely site for subsequent adsorption.

Note that the observed deviations from the linearity in the Scatchard plots of MON adsorption onto the six bio-adsorbents here studied are attributed to different affinities of the binding sites toward MON molecules. Consequently (although needing complementary studies, such as FTIR analyses to make it evident), it could be considered that the carboxyl groups (which had relatively low pk_a_ values) of adsorbent materials (both fibers and barks), especially those adsorbing more MON, and exhibiting suitable conformations for antibiotic binding, may potentially intervene in the main high-affinity (strong) binding sites, whereas phenolic groups exhibiting relatively high pK_a_ values are assumed to be the main low-affinity (weak) binding sites (Figure S2, Supplementary Material).

Furthermore, as shown in Table S3 (Supplementary Material), the values of the binding constants (K_b_) and the maximum capacities (Q_m_^S^) of high- and low- affinity levels were separately calculated. According to Table S3 (Supplementary Material), it can be seen that the obtained K_b_ and Q_m_^S^ values were very close to those calculated for the Langmuir model (except for cactus and palm fibers). Based on these results, the adsorption of MON onto the three natural barks and alfa fiber was primarily attributed to the high-affinity binding sites. Conversely, the interactions between the MON molecules and the cactus/palm fibers were governed by the low-affinity binding sites, and it was believed that the binding involving the complex formations had actually occurred through complex formation [50]. Thus, the low-affinity binding is caused by the complex formation, whereas the high-affinity binding is associated with the ion exchange mechanism in the MON adsorption onto natural barks and alfa fiber along with the other mechanisms cited above.

### 4.4. MON Desorption

Regarding the results shown in Figure 4, the MON desorption values obtained for the three barks were lower than those reported in a previous study for clarithromycin using pine bark as sorbent, where percentages of 15% were reached when adding 100 µmol L^−1^ [91].

The influence of the increase in the initial MON concentration added on rising desorption was clearly observed for the bio-adsorbents with the lowest removal efficiency (cactus and palm fiber), while the relation was less marked in the case of zean oak bark (Figure 4). However, for the most efficient bio-adsorbents (both eucalyptus and acacia barks, and alfa fiber), this is not the case, as a slight decrease in the desorption percentage is observed when the antibiotic dose is increased. The lower desorption scores of the latter bio-adsorbents may be related to some of their physicochemical characteristics, especially their pH and OM content, which would facilitate dissociation of organic functional groups, allowing a strong binding of the antibiotic that hinders its desorption. Similar conclusions were reported by Hu et al. [92] when studying sulfadiazine and sulfamethoxazole in different agricultural soils. Additionally, Jeong et al. [93] indicated that adding softwood and hardwood biochar as low-cost adsorbents to soils considerably decreased the desorption of the macrolide antibiotic tylosin. In the current research, we found lower desorption percentages for the three barks and alfa fiber compared to those previously reported for cefuroxime desorption from eucalyptus leaves and pine bark [59] or compared to sulfonamides from different agricultural soils [25]. This would encourage additional in-depth studies focused on using the bio-adsorbents here investigated as soil amendments.

Considering the hysteresis values (HI, Table 3), the scores were relatively high for most of the bio-adsorbents here studied, reflecting a slow desorption process [86,94], except for cactus and palm fibers.

In relation to fitting of the desorption experimental data to different models (Table 4), in the Temkin equation the bt_(des)_ values (which were in the range 0.028–0.532) were consistently lower than those of the bt_(ads)_ parameter (0.231–6.765), except for both palm and cactus fibers, suggesting the low reversibility of the bonds [95]. This fact complements the information derived from Sips’s K_S(des)_ values, which were typically lower than those of K_S(ads)_ (Table 2). Meanwhile, the q_m_ values obtained from the Sips model (ranging between 6.0 and 175.7 µmol kg⁻^1^) were consistent with those observed for the real desorption data (oscillating between 9.4 and 172.9 µmol kg⁻^1^) obtained in the current study (Figure 4). In contrast, the q_m_ values of the Langmuir model (oscillating between 178.1 and 443.2 µmol kg⁻^1^) were clearly higher than those observed for the real desorption data (Table 4). In fact, a good fit was not found for this model, with R^2^ not exceeding values of 0.745 in all cases (Table 4). In the same way, the desorption data did not fit either the Freundlich model or the Linear model. Additional research in this field, as well as in other related aspects of soil and environment sciences, would be a must for environmental and public health protection, and also for promoting recycling, crop sustainability, and the circular economy [96,97].

Further in-depth research is required to clarify the specific mechanisms involved in both the MON adsorption and desorption processes on the studied sorbent materials. This research is planned for the near future and will involve complementary analytical techniques, such as FTIR analysis of samples before and after adsorption and desorption, as well as other specialized analyses currently being refined and implemented. Additionally, super-computational modeling will be employed to investigate the interactions between the pollutants and the sorbents across a range of environmental conditions [51].

## 5. Conclusions

Eucalyptus and acacia barks, along with alfa fiber, were the most efficient bio-adsorbents among those tested in the current research for MON retention (with adsorption always >76.6%). These three bio-adsorbents have the lowest pH values, as well as the highest organic matter contents and eCEC scores, with higher levels of exchangeable Na and Ca (and less exchangeable K and Mg). The Temkin model was the most appropriate for explaining MON adsorption onto the six bio-adsorbents (R^2^ ≥ 0.968), indicating the relevance of chemisorption processes based on strong electrostatic interactions between positive and negative charges. Furthermore, the good fit of the Langmuir model to the adsorption experimental data (R^2^ ≥ 0.934) suggests the dominance of chemical and monolayer adsorption on surfaces with finite, energetically equal sites, as evidenced by the significant decrease in adsorption efficiency observed at higher MON concentrations added. The bio-adsorbents that present the highest MON adsorption (the three barks and alfa fiber), desorb a low proportion of the previously retained antibiotic, indicating a low reversibility for the process. In relation to this, the values of the hysteresis index for these bio-adsorbents were clearly lower than those obtained for the rest of the materials, with a greater tendency towards desorption of the antibiotic by cactus and palm fibers. Valorizing both eucalyptus and acacia barks, along with alfa fiber, would lead to a more efficient use of these by-products, potentially offering environmental and economic benefits with regards to environmental remediation in MON-polluted compartments. For the future, it would be interesting to perform more in-depth studies, in a variety of experimental and environmental conditions, focused on the removal of MON and other anticoccidials, as well as of other emerging pollutants, using the low-cost bio-adsorbents here assessed, which could be raw or modified when justified. This would be in line with the promotion of recycling and sustainability, as well as with environmental and public health protection.

## Figures and Tables

**Table 1:** Chemical characteristics of the different bio-adsorbents, with average values (n = 3) and coefficients of variation always <5%. EC: electrical conductivity (in dS m^−1^); (H%): Moisture content (in percentage); DM: Dry matter content (%): P: Porosity (in percentage); As: Ash content (%); VM: Volatile matter content (%); BD: Bulk density (in g cm^−3^); RD: Real density (in g cm^−3^); SI: Swelling Index (in percentage); OM: organic matter content (%); OC%: Organic carbon content (in percentage); X_e_: exchangeable cations (Al, Ca, K, Mg, and Na, expressed in cmol_c_ kg^−1^); eCEC: effective cation exchange capacity (expressed in cmol_c_ kg^−1^).

	Alfa Fiber	Cactus Fiber	Palm Fiber	Acacia Bark	Eucalyptus Bark	Zean Oak Bark
pH_w_	5.1	7.4	5.5	4.9	5.4	5.7
pH_KCl_	4.7	7.6	6.9	4.2	5.1	4.8
pH_PZC_	6.6	6.2	4.3	7.1	7.4	5.8
EC	21	204	818	19.7	4.2	5.9
H%	9.3	6.2	5.4	10.7	11.5	7.0
DM	90.7	93.8	94.6	89.3	88.5	93.0
P	86.6	48.0	41.6	62.8	65.7	57.5
As	2.45	3.34	4.36	1.22	1.42	1.91
VM	97.55	96.66	95.64	98.58	98.58	98.09
BD	1.28	0.96	0.81	1.42	1.53	1.1
RD	1.65	1.23	1.14	1.86	1.97	1.43
SI	2.56	1.43	1.08	2.82	2.96	1.84
OM	40.72	21.76	18.84	49.21	50.25	29.85
OC%	19.54	10.44	9.04	23.62	24.12	14.32
Al_e_	0.11	0.05	0.05	0.18	0.11	0.09
Ca_e_	6.74	2.04	1.97	7.44	7.98	4.22
K_e_	2.22	3.04	2.96	2.02	2.07	2.79
Mg_e_	1.57	3.06	2.99	1.03	0.86	2.81
Na_e_	2.32	1.05	0.96	3.77	4.04	1.12
eCEC	13.97	9.26	8.55	14.45	15.08	11.04
Paricle size (%)						
0.075–0.1 mm	86.17	52.28	66.71	26.14	31.30	39.70
0.05–0.075 mm	11.68	30.61	15.77	68.43	65.45	53.40
0.05–0.02 mm	2.15	10.73	13.47	4.16	3.25	6.78
<0.02 mm	--	6.38	4.05	1.27	--	1.20

**Table 2:** Values corresponding to the fitting of the experimental data (referred to MON adsorption onto the six bio-adsorbents) to the parameters of the Freundlich, Langmuir, Linear, Sips, and Temkin models. K_F_ (L^n^ µmol^1−n^ kg^−1^); K_L_ (L kg^−1^); q_m_ (µmol kg^−1^); K_d_ (L kg^−1^); K_s_ (L kg^−1^); K_t_ (L g^−1^); b_t_ (J/mol). R^2^: coefficient of determination; -: error too high for fitting.

		Alfa Fiber	Cactus Fiber	Palm Fiber	Acacia Bark	Eucalyptus Bark	Zean-Oak Bark
Freundlich model	K_F_	227.7	116.1	57.4	331.1	470.4	195.5
	Error	40.1	9.8	19.1	93.1	101.2	6.4
	n	1.415	0.212	0.236	2.484	2.685	0.458
	Error	0.05	0.03	0.01	0.11	0.28	0.09
	R^2^	0.762	0.826	0.817	0.723	0.741	0.842
Langmuir model	K_L_	0.14	0.06	0.04	0.27	0.31	0.08
	Error	0.01	0.00	0.01	0.02	0.03	0.00
	q_m_	957.9	835.8	809.9	987.2	1046.1	527.9
	Error	97.4	80.7	60.6	107.5	140.3	37.48
	R^2^	0.997	0.962	0.995	0.972	0.989	0.934
Linear model	K_d_	28.85	5.04	2.29	114.9	167.8	6.46
	Error	5.52	0.49	0.28	30.4	34.9	0.3
	R^2^	0.634	0.622	0.611	0.631	0.578	0.601
Sips model	K_s_	3.21	1.00	0.18	4.77	6.2	1.85
	Error	0.13	0.01	0.00	0.93	1.1	0.05
	n	1.523	0.270	0.173	2.137	2.754	0.441
	Error	0.13	0.1	0.007	0.24	0.22	0.02
	q_m_	925.6	474.5	336.1	969.3	925.6	704.6
	Error	10.3	32.1	23.3	47.8	35.1	15.5
	R^2^	0.944	0.873	0.869	0.925	0.932	0.884
Temkin model	K_t_	2.71	0.64	-	3.84	4.21	-
	Error	0.004	0.00	-	0.34	0.001	-
	b_t_	4.772	0.344	0.231	5.022	6.765	2.522
	Error	0.8	0.05	0.09	0.00	1.33	1.53
	R^2^	0.989	0.979	0.968	1.00	1.00	0.982

**Table 3:** Hysteresis index (HI) corresponding to the desorption of MON from the six bio-adsorbents, and for each of the initial concentrations of the antibiotic added.

MON Concentration Added (µmol L^−1^)	Hysteresis Index (HI)					
	Alfa Fiber	Cactus Fiber	Palm Fiber	Acacia Bark	Eucalyptus Bark	Zean Oak Bark
5	0.852	0.977	0.982	0.904	0.869	1
10	0.907	0.956	0.961	0.914	0.895	1
20	0.935	0.855	0.918	0.921	0.903	0.996
40	0.973	0.390	0.748	0.932	0.914	0.943
80	0.979	−0.221	−0.248	0.938	0.919	0.914
100	0.985	−1.546	−1.281	0.957	0.936	0.824
Average values	0.938	0.235	0.429	0.928	0.906	0.946

**Table 4:** Values corresponding to the fitting of the experimental data (referred to MON desorption from the six bio-adsorbents) to the parameters of the Freundlich, Langmuir, Linear, Sips, and Temkin models. K_F_ (L^n^ µmol^1−n^ kg^−1^); K_L_ (L kg^−1^); q_m_ (µmol kg^−1^); K_d_ (L kg^−1^); K_s_ (L kg^−1^); K_t_ (L g^−1^); b_t_ (J/mol). R^2^: coefficient of determination; -: error too high for fitting.

		Alfa Fiber	Cactus Fiber	Palm Fiber	Acacia Bark	Eucalyptus Bark	Zean Oak Bark
Freundlich model	K_F_	0.627	-	17.638	2.685	-	-
	Error	0.263	-	2.143	0.519	-	-
	n	0.144	-	0.045	2.732	3.112	0.769
	Error	0.021	-	0.015	0.331	0.301	0.180
	R^2^	0.737	-	0.892	0.719	0.741	0.868
Langmuir model	K_L_	0.204	0.009	0.049	0.06	0.121	0.070
	Error	0.001	0.00	0.022	0.03	0.042	0.001
	q_m_	281.36	443.21	324.65	270.58	254.36	178.12
	Error	32.02	97.46	65.13	104.70	123.23	54.82
	R^2^	0.653	0.573	0.465	0.705	0.713	0.745
Linear model	K_d_	0.641	6.210	0.955	0.423	0.418	0.162
	Error	0.182	0.521	0.026	0.072	0.068	0.029
	R^2^	0.625	0.752	0.832	0.721	0.789	0.727
Sips model	K_s_	0.875	-	-	-	-	0.052
	Error	0.00	-	-	-	-	0.0021
	n	0.872	0.445	1.972	0.582	0.673	0.341
	Error	0.00	0.012	0.052	0.00	0.00	0.001
	q_m_	59.60	64.211	175.73	25.195	28.012	6.055
	Error	11.00	15.022	23.06	19.540	10.332	2.013
	R^2^	0.918	0.950	0.934	0.932	0.987	0.995
Temkin model	K_t_	0.381	1.292	2.887	1.022	1.307	0.077
	Error	0.049	0.011	0.153	0.142	0.062	0.010
	b_t_	0.405	0.532	0.112	0.311	0.285	0.028
	Error	0.155	0.213	0.003	0.101	0.031	0.00
	R^2^	0.998	0.989	0.984	1.00	1.00	0.993

## Data Availability

Experimental data could be provided after specific request and after receiving authorization from the funding agency.

## Supplementary Materials

The following supporting information can be downloaded at: https://www.mdpi.com/article/10.3390/toxics12080606/s1, Table S1: Main physicochemical characteristics of monensin (MON). K_oc_: organic carbon partition coefficient; K_ow_: octanol-water coefficient of partition; Kc: equilibrium constant. Table S2: Variation of pH values in aqueous media containing MON and the studied bio-adsorbents. Table S3: Scatchard parameters for monensin adsorption onto the six studied bio-adsorbents. Figure S1: HPLC example chromatograms. Figure S2: Scatchard plots derived for adsorption data obtained at natural pH for the six studied biomaterials. Figure S3: pH_PZC_ of the six bio-adsorbents (T = 25 ± 2 °C). Figure S4: Effect of the initial pH on MON adsorption onto the bio-adsorbents used. Figure S5: Illustration of the pseudo-cyclic conformation of the MON-Na complex (adapted from [38]. References [98,99,100,101,102,103,104] are cited in the supplementary materials.
