# Supplementary material for: Removal of the Highly Toxic Anticoccidial Monensin Using Six Different Low-Cost Bio-Adsorbents
Source: Toxics. 2024 Aug 19;12(8):606. doi: 10.3390/toxics12080606 (PMC11360468; doi:10.3390/toxics12080606)
Supplement: Supplementary file 1 [file toxics-12-00606-s001.zip › toxics-3138506-supplementary.pdf]

Supplementary material

Removal of the Highly Toxic Anticoccidial Monensin Using Six Different Low-Cost Bio-Adsorbents

Samiha Hamdi[a,b,c], Manel Issaoui [a,c] , Sonia Hammami[c], Ainoa Míguez-González[b], Raquel Cela-Dablanca[b], Ana Barreiro[b,*], Avelino Núñez-Delgado[b], Esperanza Álvarez-Rodríguez[b], María J. Fernández-Sanjurjo[b]

[a]Department of Biotechnology, Faculty of Science and Technology of Sidi Bouzid, University of Kairouan, 9100 Sidi Bouzid, Tunisia

[b]Department of Soil Science and Agricultural Chemistry, Engineering Polytechnic School,

University of Santiago de Compostela, 27002 Lugo, Spain

[c]Laboratory of Nutrition – Functional Foods and Health (NAFS)-LR12ES05, Faculty of medicine, University of Monastir, Avenue Avicenne 5019 Monastir, Tunisia

***** Correspondence author: Ana Barreiro (ana.barreiro.bujan@usc.es)

Table S1. Main physicochemical characteristics of monensin (MON). K_{oc}: organic carbon partition coefficient; K_{ow}: octanol-water coefficient of partition; Kc: equilibrium constant.

Molar weight (g mol^{-1})	Chemical formula	Solubility in water (mg L^{-1})	pk$_a$	Stability	Produced by	Log k$_{oc}$	Log k$_{ow}$	Log (K$_c$)
671[a]	$C_{36}H_{62}O_{11}$	4.8 to 8.9[a]	4.5[d]	Unstable in acidic condition, stable in alkaline condition[a]	*Streptomyces cinnamonensis*[c]	> 5.6[b]	> 6.3[b]	−1.0 in water[d] −1.4 in methanol[d]

[a][15]; [b][98]; [c][70]; [d][60].

1. Characterization of the bio-adsorbents

The pH (pH$_w$) and electrical conductivity (EC) values in water, as well as the pH$_{KCl}$ value (in 0.1 M KCl solution), were measured using a combined glass electrode (pH-meter model 2001, Crison, Barcelona, Spain), at a 1:2.5 solid:liquid ratio, with contact times of 10 min for pH$_w$ and EC and 120 min for pH$_{KCl}$.

To investigate the relationship between surface charges of the studied biosorbents and the solution pH, the determination of pH$_{PZC}$ was carried out. Sodium hydroxide (NaOH) and hydrochloric acid (HCl) solutions were used to adjust the pH of 0.01 M sodium chloride (NaCl) solutions to a range of 1–12. For each pH level, 50 mL of NaCl solution were mixed with 0.5 g of each biosorbent and stirred at 350 rpm for 24 h. The pH $_{final}$ (pH$_f$) of each solution was then measured using a CRISON 2001 pH-meter (Crison, Barcelona, Spain) [55, 101]. The pH$_{PZC}$ is considered as the point where the curve of pH$_{final}$ versus pH$_{initial}$ (pH$_i$) intersects the straight line corresponding to pH$_{initial}$ = pH$_{final}$; at this pH, the net surface charge of the biosorbent is zero (Fig. S1) [66].

The moisture content (H%), the porosity (P), the ash content (%), and the real density (RD) were determined according to Della et al. (2023) [66], while the bulk density (BD) was measured as reported in Qlihaa et al. (2016) [102]. Briefly, the moisture content (H%) was

determined by drying a specific mass of each sorbent material (m_0=1g) in an oven at 105 °C for 24 h until their weights stabilized (m_1), and then it was calculated using the following formula: H% = [$m_0 - m_1 / m_0$] × 100. Thus, the dry matter (DM) content (%) is defined as the total mass of the bio-adsorbent minus the moisture content.

The bio-adsorbents' porosity (P, expressed in percentage) was considered as the ratio of the volume of voids (V_1) to the total volume of the material (V_t) (Della et al., 2023). It was measured by filling a 10 mL test tube with mass M_1 of each adsorbent being examined until it reached a volume (V_2). Subsequently, methanol was added until it reached the volume V_t (corresponding to mass m_2). The P values were then calculated as shown in the following formula:

$P = V_1/Vt = ([(m_2 – m_1) / \rho_m] – V_2) / V_t$, where V_1 is considered as the void volume (expressed in cm^3); V_2 is the volume of the solid (expressed in cm^3); V_t represents the total volume (expressed in cm^3); and ρ_m is the density of methanol (expressed in g cm^{-3}). To determine the ash (As) content (%) in the adsorbent samples, 1 g (mass W_1) of each dry sorbent material (dried previously in an oven at 60 °C) was weighed and introduced into a calcination crucible, and then placed into an oven at 600 °C for 4 h. After cooling, the crucibles were weighed again to obtain the mass W_2. The calculation of ash content, which serves as an indicator of the mineral content present in the material, was performed as follows: As (%) = (W_2/ W_1) × 100.

After calculating the As content in all biomaterials, the volatile matter (VM) content is typically concluded as a percentage of the total weight (100%), excluding the ash content (in percentage). Using a pycnometer, the bulk density (BD) of the studied samples was determined and then calculated as the volume of a mass of each bio-adsorbent sample (V_m) divided by the mass of the same volume of water (M_w) , as described by the formula [102] : BD = V_m/ M_w. Hence, the real density was defined by introducing a specific quantity of each dehydrated adsorbent to be analyzed (m_0) into a weighed pycnometer, which was filled with methanol and weighed in a

further step. Knowing the tare weight and the methanol density ρ_m (0.792 g cm^{-3}) allows the determination of the real density, using the following equation: RD= $(m_0 \times \rho_m)/(m_1 - m_2)$.

To measure the swelling index (SI, in percentage) of each biosorbent sample, approximately 0.5 g (W_1) of each was allowed to swell in 100 mL xylene using a rotary shaker at 50 rpm (SIR EM-SA, motor: 220V, N1: 2700 rpm, N2: 48 rpm) in the dark and at room temperature (25 ± 2 ºC) for 24 h. The insoluble portion was then filtered and weighed (W_2). After that, the sample was oven-dried at 130 ºC for 24 h to remove excess solvent. Once completely dry, the sample was reweighed (W_3). The swelling index (SI) was calculated as follows ([103, 104]: SI (%) = $(W_3 - W_1)/W_3$ x100

The measurement of the organic matter (OM) content was performed by means of the loss on ignition (LOI) method, putting 1 g of each of the adsorbents into a previously weighted porcelain crucible, which was placed in an oven (with maximum temperature =1200 °C), with a gradual increase in temperature until it reached 550 °C, then removing and placing it in a desiccator to cool, followed by weight measurement; the loss on ignition values was given by the formula: LOI (%) = 100× (n2-n3)/(n2-n1), where n1 represents the weight of the empty ceramic crucible, n2 is the initial weight of the ceramic crucible with the sample powder, and n3 is the total mass (the ceramic crucible and the sample powder). The organic carbon (OC) content in each tested biomaterial can be deducted from the obtained values of the OM content using the following equation [99]: OC (%) = 0.48×OM (%). Chemical analyses, involving the determination of exchange cations (Al_e, Ca_e, Mg_e, Na_e, and K_e), were carried out by extracting them from the bio-adsorbents using a 1 M NH_4Cl extractant [40], in a 1:10 adsorbent:solution ratio, for 12 h; atomic adsorption/emission spectrophotometry, performed using an Analyst 200 from PerkinElmer (USA) equipment, was employed to quantify the exchangeable cations, adding 1% of $LaCl_3$ to avoid interferences; the sum of these chemical elements was made to obtain the effective cation exchange capacity (eCEC) [37].

For the six bio-adsorbents, the range of particle sizes were determined using the wet sieving method (at and below 100 µm, using sieves of 100, 75, 50, and 20 µm mesh). Out of all the fractions obtained after the sieve analysis, the different sample fractions of > 100 µm, 75-100 µm, 50-75 µm, 50-20 µm, and < 20 µm were obtained. The percentage of aggregate passing through each sieve was determined by calculating the percent of samples retained in each sieve, which was done by using the following equation: % Retained = (W_{Sieve}/ W_{Total}) ×100, where W_{Sieve} was the weight of aggregate in the sieve and W_{Total} was the total weight of the aggregate [100].

After subjecting the powdered fibers and barks to sieve analysis, particles larger than 100 µm were not taken up for the experiment. Therefore, all other fractions were selected.

2. Adsorption-desorption experiments

In the present study, the MON concentrations used ranged from 5 to 100 µmol L^{-1}. The experimental conditions considered were adsorbent weigh (0.5 g), adding 10 mL of the MON solutions, contact time (48 h, in the dark, to prevent MON photodegradation, as the stability of these molecules would be affected by light exposure, especially under ultraviolet radiation), and MON concentration (5-100 µmol L^{-1}). These factors were selected by taking into account the adsorption capacity of some forest bio-adsorbents described in the literature, and also based on previous unpublished experiments performed for our biomaterials, depending on their physicochemical characteristics. Additionally, the experiments were carried out at room temperature (25 ± 2 °C).

Briefly, after the adsorbent amounts were added to the antibiotic solutions (0.5 g to 10 mL), the suspensions underwent 48 hours of agitation on a rotary shaker (SIR EM-SA, motor: 220V, N1: 2700 rpm, N2: 48 rpm) at 50 rpm. Subsequently, the agitated suspensions underwent centrifugation (4000 rpm, 15 minutes) and filtration using 0.45 µm nylon syringe filters (Fisher

Scientific, Madrid, Spain). pH measurements were conducted with a pH meter (Crison, Barcelona, Spain).

After the adsorption phase, subsequent desorption experiments were conducted following the same procedure used for adsorption. The weighed remaining material was introduced into 10 mL of 0.005 M $CaCl_2$, and the samples were allowed to stir for 48 hours. Subsequently, they were centrifuged and filtered under the same conditions as those used for the adsorption process. In the equilibrium solution, the concentration of the MON antibiotic was determined, analogously to that indicated in the adsorption tests. Three replicates were used for each adsorption-desorption test.

3. Pre-quantification procedure before MON measurement in the samples from the adsorption-desorption tests

Firstly, 100 µL of trichloroacetic acid (TCA) were added to 700 µL of each sample from the adsorption/desorption phases, and the mixtures were rapidly agitated [39]. . Following a 10-minute incubation period, 200 µL of 2,4-dinitrophenol (DNP) acid were added, and the samples were agitated once more before being placed in an oven at 50 °C for 20 minutes, following the procedure outlined by Nebot et al. (2011) [39]. Finally, the concentrated MON antibiotic was quantified in the filtered (through 0.45 µm) and previously prepared supernatants using HPLC-UV Thermo Scientific Ultimate 3000 equipment, model LPG 3400 SD (Thermo-Scientific, USA).

4. HPLC equipment

The analysis of the antibiotic concentrations was conducted using the HPLC-UV Thermo Scientific Ultimate 3000 equipment, specifically the LPG 3400 SD model (Thermo-Scientific, USA). It is equipped with a quaternary pump and an auto-sampler. Chromatographic separations were performed on a C18 analytical column (150 mm length, 4.6 mm internal diameter, 5 µm particle size) from Phenomenex (Madrid, Spain), along with a pre-column (4 mm length, 3 mm internal diameter, 5 µm particle size) packed with the same material as the column [41]. Chromatograms obtained during the analysis were processed using Chromeleon software version 7 (Thermo Fisher Scientific, Madrid, Spain).

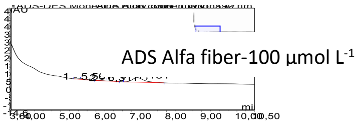

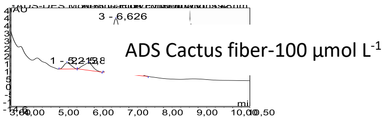

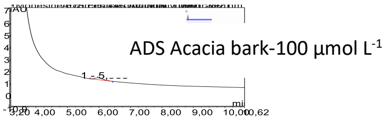

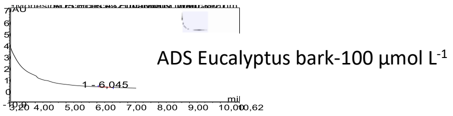

Figure S1. HPLC example chromatograms.

5. Further details from the experiments

These additional details are presented in Table S2 and S3, and Figures S2, S3, S4, and S5 below.

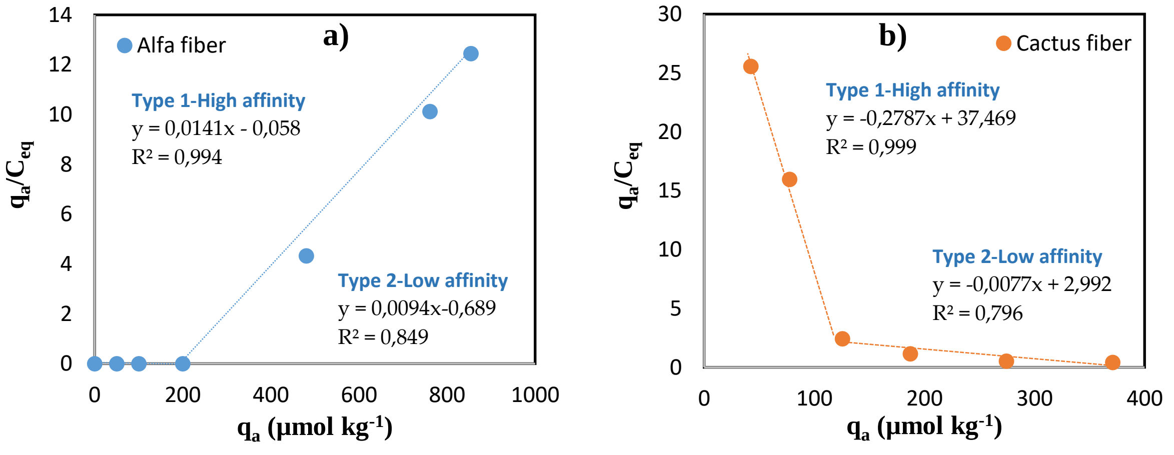

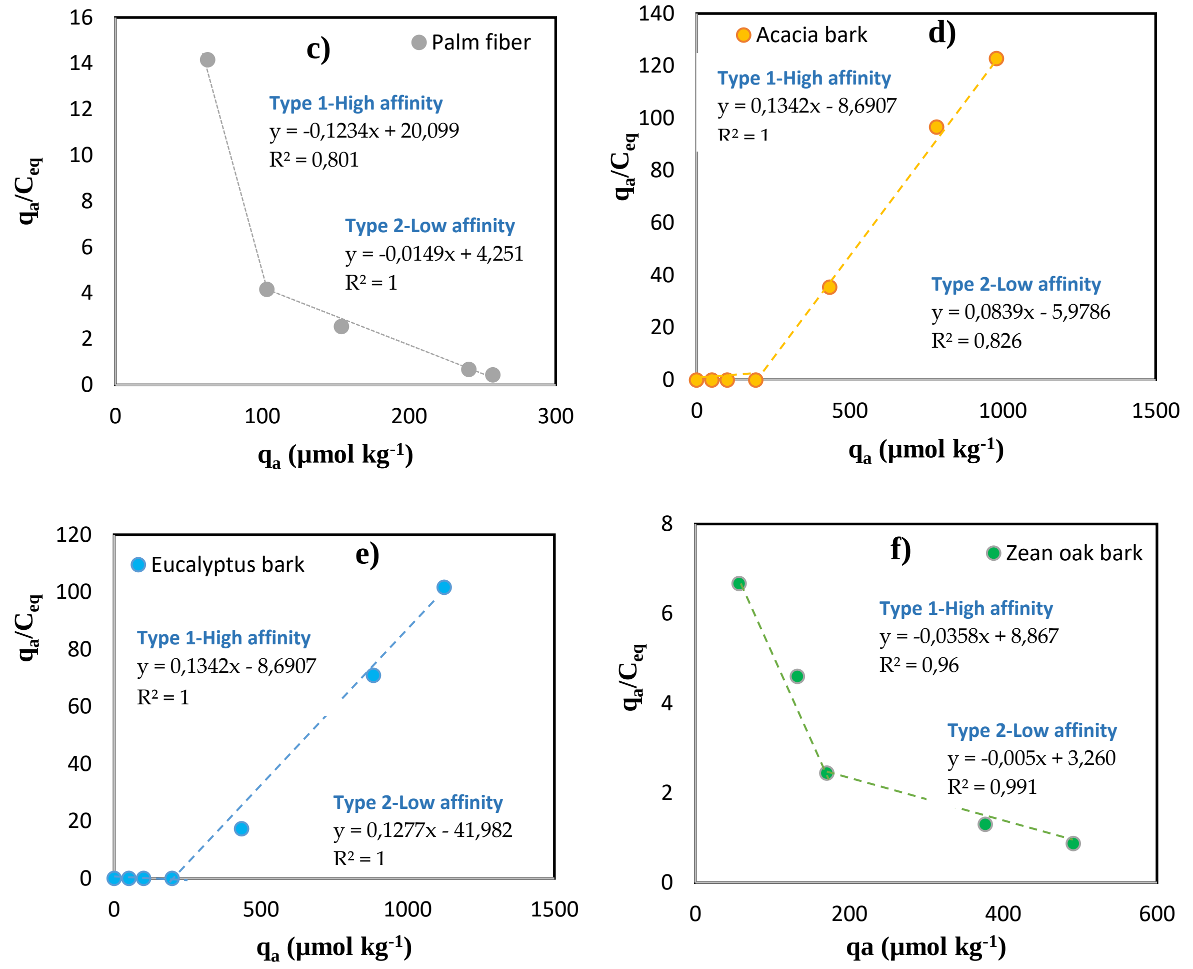

Figure S2. Scatchard plots derived for adsorption data obtained at natural pH for the six studied biomaterials: the three natural fibers (a, b, c) and barks (d, e, f). q_a: the amount of the MON adsorbed (in µmol kg^{-1}); C_{eq}: the concentration of MON in the equilibrium. Adsorption tests conditions: 0.5 g of sorbent material with 10 mL of 0.005 M $CaCl_2$ solutions containing from 0 to 100 µmol L^{-1} of MON, shaking for 48 h in the dark and at 25 ± 2 ºC at 50 rpm, then centrifuging (4000×g) and filtering by 0.45 µm before HPLC quantification.

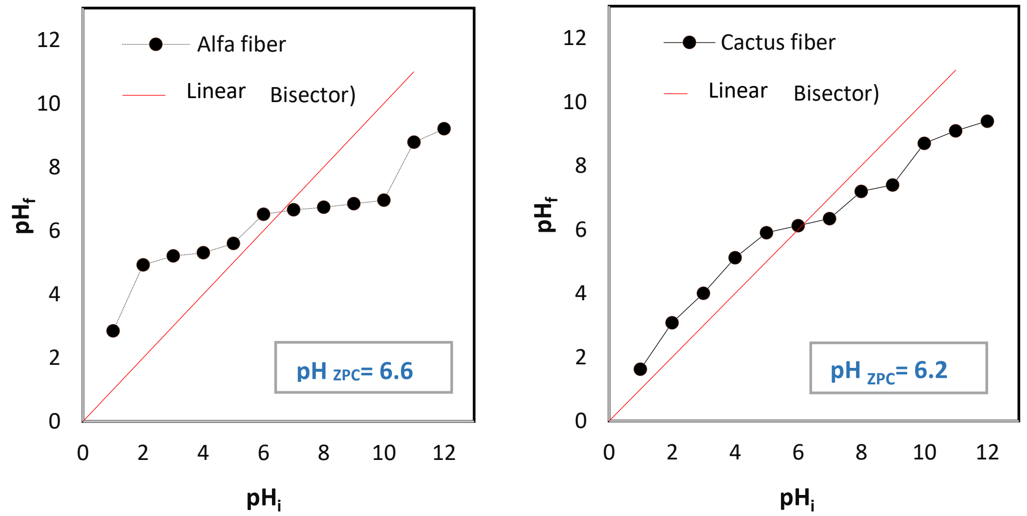

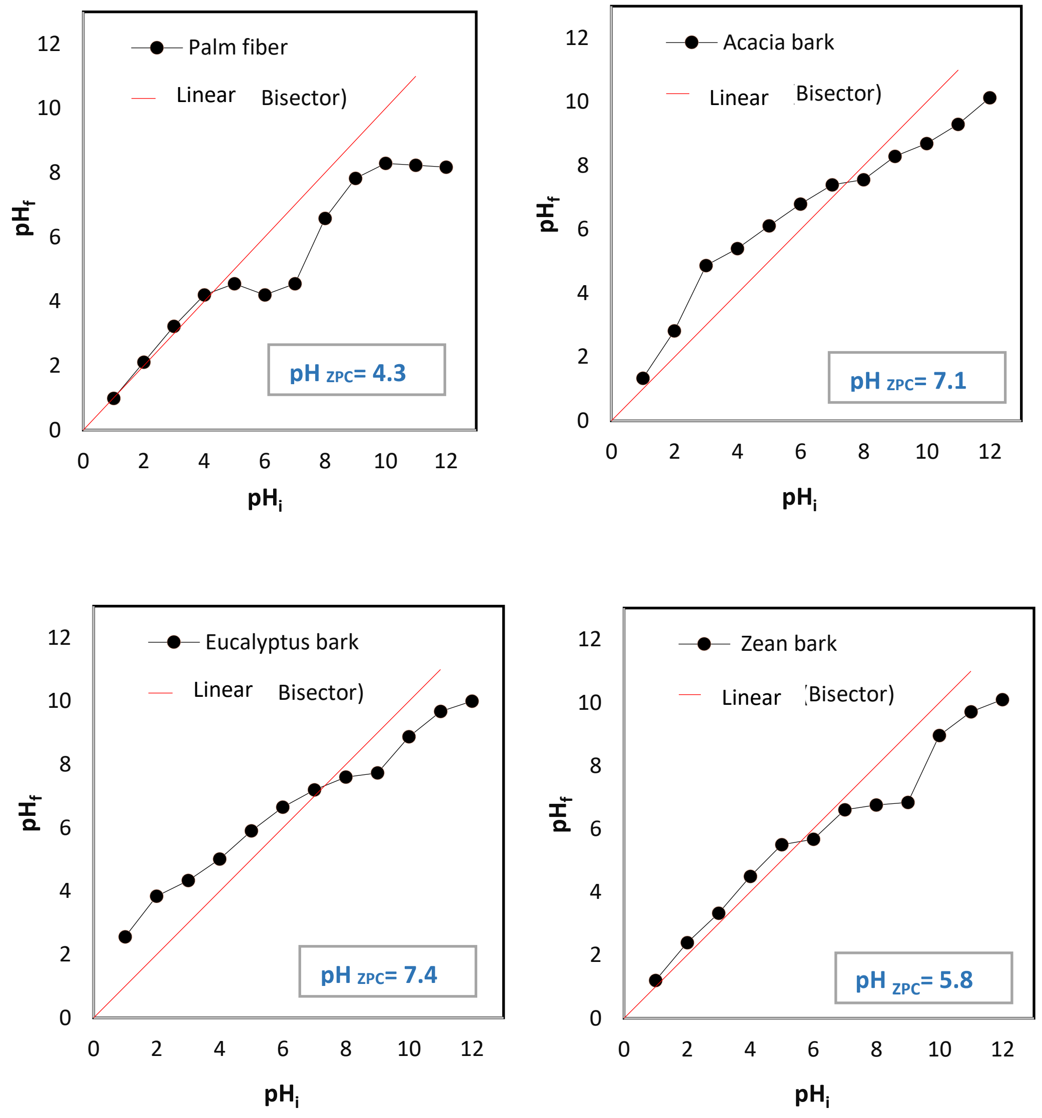

Figure S3. pH_{PZC} of the six bio-adsorbents (T = 25 ± 2 °C).

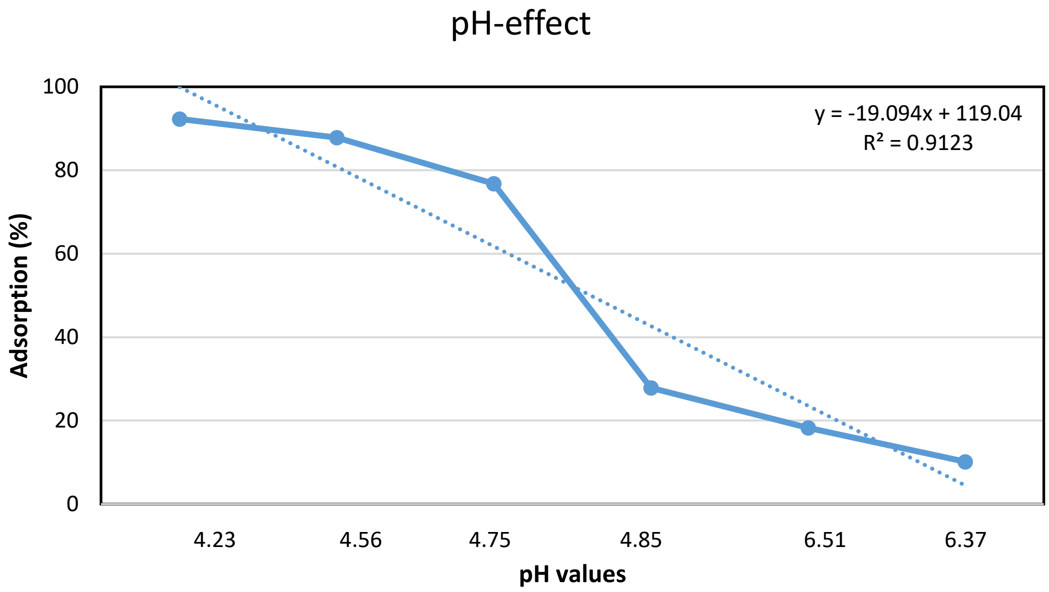

Figure S4. Effect of the initial pH on MON adsorption onto the bio-adsorbents used.

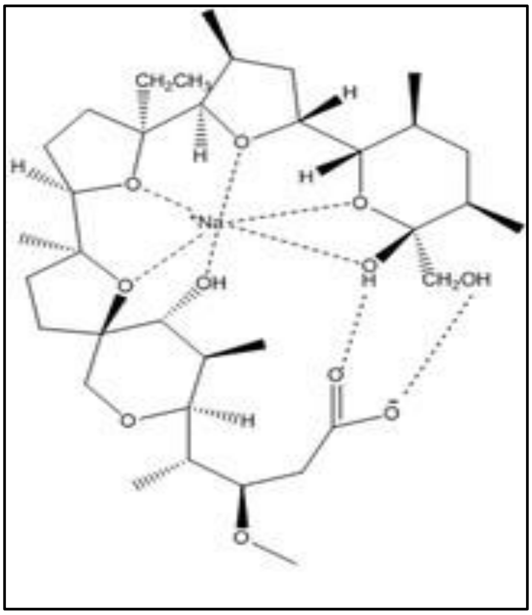

Figure S5. Illustration of the pseudo-cyclic conformation of the MON-Na complex (adapted from Lopes et al., 2002 [38]).

Table S2. Variation of pH values in aqueous media containing MON and the studied bio-adsorbents.

	Bio-adsorbent	pH ads (CaCl2)		
		Min	Max	Mean
MON +	Alfa fiber	4.75	5.02	4.88
	Cactus fiber	6.37	6.92	6.64
	Palm fiber	6.51	6.77	6.64
	Acacia bark	4.56	4.81	4.9
	Eucalyptus bark	4.23	4.62	4.83
	Zean oak bark	4.85	4.98	9.69

pH_{ads}: pH of the aqueous media containing MON and different bio-adsorbents during the adsorption process. Mean values correspond to three repetitions, with coefficients of variation always lower than 5%.

Table S3. Scatchard parameters for monensin adsorption onto the six studied bio-adsorbents.

	**Affinity level**	**Alfa fiber**	**Cactus fiber**	**Palm fiber**	**Acacia bark**	**Eucalyptus bark**	**Zean oak bark**
$\mathbf{Q_m^S}$	High affinity (H)	867.91	126.76	112.3	986.159	1138.53	496.332
	Low affinity (L)	207.09	849.67	772.89	195.122	200.34	128.91
$\mathbf{K_b}$	High affinity (H)	0.165	0.293	0.245	0.298	0.332	0.092
	Low affinity (L)	0.586	0.063	0.0514	0.828	0.772	0.467

References

15. Hussain, SA.; Prasher, SO.; Patel, RM. Removal of ionophoric antibiotics in free water surface constructed wetlands. Ecol Eng 2012, 41, 13-21. https://doi.org/10.1016/j.ecoleng.2011.12.006

37. Fox, RL., Kamprath, EJ., 1970. Phosphate sorption isotherms for evaluating the phosphate requirements of soils. Soil Science Society of America Journal 34, 6, 902-907.https://doi.org/10.2136/sssaj1970.03615995003400060025x

38. Lopes, NP., Stark, CB., Gates, PJ., Staunton, J., 2002. Fragmentation studies on monensin A by sequential electrospray mass spectrometry. Analyst 127,4,503–506. https://doi.org/10.1039/B110412H

39. Nebot, C., Iglesias, A., Regal, P., Miranda, JM., Fente, C., Cepeda., A., 2011. A sensitive and validated HPLC–MS/MS method for simultaneous determination of seven coccidiostats in bovine whole milk. Food Control 27, 1, 29-36. https://doi.org/10.1016/j.foodcont.2012.02.012

40. Peech, M., 1947. Methods of soil analysis for soil-fertility investigations, U.S. Dept. Agr. Circ. 757, 7-11.

41. Rodríguez-López, L., Santás-Miguel, V., Cela-Dablanca, R., Núñez-Delgado, A., Álvarez-Rodríguez, E., Pérez-Rodríguez, P., Arias-Estévez, M., 2022. Ciprofloxacin and Trimethoprim Adsorption/Desorption in Agricultural Soils. International Journal of Environmental Research and Public Health 19, 8426. https://doi.org/10.3390/ijerph19148426

55. Karoui, S., Ben Arfi, R., Fernández-Sanjurjo, MJ., Nuñez-Delgado, A., Ghorbal, A., Álvarez-Rodríguez, E., 2021. Optimization of synergistic biosorption of oxytetracycline and cadmium from binary mixtures on reed-based beads: modeling study using Brouers-

Sotolongo models, Environmental Science and Pollution Research 28, 46431–46447. https://doi.org/10.1007/s11356-020-09493-7

60. Sun, P.; Pavlostathis, SG.; Huang, CH. Estimation of environmentally relevant chemical properties of veterinary ionophore antibiotics. Environ Sci Pollut Res 2016, 23, 18353–18361. https://doi.org/10.1007/s11356-016-7029-y

66. Della, KD., Henini, G., Laidani, Y., 2023. A biosorbent material from brahea edulis palm leaves – application to amoxicillin adsorption. Cellulose Chemistry and Technology 57,7-8, 903-910. http:// doi.org//8(2023)/p.903-910

70. Hussain, SA.; Prasher, SO. Understanding the Sorption of Ionophoric Pharmaceuticals in a Treatment Wetland. Wetlands 2011, 31, 563–571. https://doi.org/10.1007/s13157-011-0171-x

98. Anonymous. Opinion of the Scientific Panel on Additives and Products or Substances used in Animal Feed on a request from the Commission on the safety and the efficacy of product “BIO-COX 120G” as feed additive in accordance with Council Directive 70/524/EEC. EFSA J 2004, 75, 1-51

99. Hamza, S., Saad, H.,Charrier, B., Ayed, N., Charrier-El Bouhtoury, F., 2013. Physico-chemicalcharacterization of Tunisian plant fibers and its utilization as reinforcement forplaster based composites. Industrial Crops and Products 49, 357-365.https://doi.org/10.1016/j.indcrop.2013.04.052

100. Jaishankar, M., Mathew, BB., Shah, MS., Murthy TPK., Gowda, KRS., 2014. Biosorption of Few Heavy Metal Ions Using Agricultural Wastes, Journal of Environment Pollution and Human Health, 2, 1, 1-6. 10.12691/jephh-2-1-1

101. Larous, S., Meniai, AH., 2016. Adsorption of diclofenac from aqueous solution using activated carbon prepared from olive stones. International Journal of Hydrogen Energy 41, 10380–10390. https://doi.org/10.1016/j. ijhydene.2016.01.096

102. Qlihaa, A., Dhimni, S., Melrhaka, F., Hajjaji, N., Srhiri, A., 2016. Physico chemical characterization of a Moroccan clay. Journal of Materials and Environmental Science 7, 5, 1741-1750.

103. Riyajan, S., Maneechay, S. 2014. Preparation and Properties of Natural Rubber Latex-GModified Cationic Polyacrylamide Copolymers and its Palm Oil Absorbent. Rubber and Composites 43,8, 264-270. https://doi.org/10.1179/1743289814Y.0000000096

104. Saramolee, P., Lopattananon, N., Sahakaro, K., 2014. Preparation and some properties of modified natural rubber bearing grafted poly(methyl methacrylate) and epoxide groups. European Polymer Journal 56, 1-10. https://doi.org/10.1016/j.eurpolymj.2014.04.008.
